# Selective Gold Ion Sorption from Iron-Containing Solution Using an Interpolymer System of Industrial Ion Exchangers

**DOI:** 10.3390/polym17202808

**Published:** 2025-10-21

**Authors:** Gulnur Dyussembayeva, Talkybek Jumadilov, Juozas Gražulevicius, Zhazira Mukatayeva, Khuangul Khimersen, Bakytgul Totkhuskyzy

**Affiliations:** 1 Bekturov Institute of Chemical Sciences, 106 Sh. Ualikhanov Str., Almaty 050010, Kazakhstan; g_gazinovna@mail.ru (G.D.); jumadilov@mail.ru (T.J.); huana88@mail.ru (K.K.); bakytgul.sakenova@mail.ru (B.T.); 2 Institute of Natural Sciences and Geography, Abai Kazakh National Pedagogical University, 13 Dostyk, Almaty 050010, Kazakhstan; 3 Department of Polymer Chemistry and Technology, Kaunas University of Technology, 73 K. Donelaičio Street, 44249 Kaunas, Lithuania; juozas.grazulevicius@ktu.lt

**Keywords:** interpolymer system, ion exchange resins, Au(I) ions, Fe(II) ions, selective sorption, desorption

## Abstract

The escalating demand for precious metals in high-tech industries and jewelry, combined with the depletion of their reserves, underscores the need for efficient methods of gold recovery from industrial effluents. An interpolymer system comprising industrial ion exchangers KU-2-8 (in H^+^ form) and AV-17-8 (in OH^−^ form) demonstrated strong selective sorption capacity for Au(I) ions from a simulated Au(I)/Fe(II) mixed solution. The optimal KU-2-8:AV-17-8 (3:3) system achieved a sorption efficiency of 97.0% for Au(I) ions after 48 h with a desorption efficiency of 98.0% using a thiourea/sulfuric acid solution. The distribution coefficient (K_d_) for Au(I) ions reached a maximum of 3233.3 mL/g at the 3:3 ratio, with a separation coefficient (β) of 40.62, indicating exceptional selectivity over Fe(II) ions. Fourier-transform infrared (FTIR) spectroscopy revealed structural changes post-sorption, including shifts in absorption bands (e.g., from 1273.5 cm^−1^ to 1292.9 cm^−1^) and the appearance of new bands (e.g., at 3171.1 cm^−1^), confirming stable ion interactions. Thermogravimetric analysis/differential scanning calorimetry (TGA/DSC) demonstrated enhanced thermal stability post-sorption, with reduced mass loss up to 100 °C. These findings highlight the KU-2-8:AV-17-8 (3:3) interpolymer system’s high selectivity, robust sorption capacity, and efficient desorption, presenting a sustainable solution for gold recovery in hydrometallurgical applications.

## 1. Introduction

The global demand for gold has surged due to its critical applications in electronics, nanotechnology, catalysis, and jewelry, driven by its unique chemical stability and conductivity [1,2,3]. With high-grade gold deposits depleting, the focus has shifted to secondary sources, such as industrial effluents, which often may contain gold in complex solutions alongside impurities such as iron, copper, nickel, etc., [4,5,6]. Gold typically exists in abovementioned solutions as stable complexes, such as Au(I) in the form of K[Au(CN)_2_], formed during cyanide leaching, coexisting with iron complexes such as K_4_[Fe(CN)_6_] [7,8,9]. The selective recovery of Au(I) ions from such mixtures remains a significant challenge in hydrometallurgy due to the chemical similarity of metal complexes and the need for environmentally sustainable and cost-effective methods [10,11,12].

Ion exchange resins, such as the strongly acidic cation exchanger KU-2-8 (H^+^) and the strongly basic anion exchanger AV-17-8 (OH^−^), are widely used for metal ions recovery due to their high exchange capacity, chemical stability, and resistance to degradation in harsh aqueous environments [13,14,15]. These polystyrene-divinylbenzene-based resins leverage functional groups—sulfonic acid for KU-2-8 (H^+^) and quaternary ammonium for AV-17-8 (OH^−^)—to facilitate selective ion binding [16,17,18]. Recent studies have highlighted the potential of interpolymer systems, which combine cation and anion exchangers, to enhance selectivity through synergistic effect under remote interaction [19,20,21]. These systems exploit mutual activation, where the interaction between oppositely charged resins induces conformational changes in polymer chains, improving ion accessibility and binding efficiency [22,23,24]. Such changes are driven by remote interaction effect, ionic strength, and pH gradients, which modulate the electrostatic environment within the resin matrix. The recovery of valuable components from solutions increasingly relies on sorption processes, which have recently garnered significant interest in research and industrial applications [25,26,27]. The recovered metals, including Au(I), and other byproducts have extensive applications across various fields, such as catalysis, pyrotechnics, coal chemistry, and more, underscoring their significant industrial and scientific importance [28,29,30,31,32,33,34].

Advancements in ion-exchange technology have focused on improving selectivity for precious metals such as Au(I), particularly in the presence of competing ions such as Fe(II). For instance, recent work has explored functionalized resins and hybrid materials to enhance sorption kinetics and capacity [35]. Interpolymer systems offer a promising alternative by combining the strengths of individual resins while mitigating limitations, such as non-specific binding or low desorption efficiency [36,37]. However, challenges remain in optimizing molar ratios and understanding the structural dynamics of polymer matrices during sorption and desorption processes [38,39,40].

This study introduces a novel application of the KU-2-8:AV-17-8 (X:Y) interpolymer system for the selective recovery of Au(I) ions in the presence of competitive Fe(II) ions, marking its first reported use in this context. The research systematically investigates sorption kinetics, desorption efficiency, and conformational changes in the polymer matrix across various molar ratios. By harnessing the synergistic interactions between the KU-2-8 (H^+^) and AV-17-8 (OH^−^) ion exchangers, this work advances the development of highly selective, efficient, and sustainable methods for Au(I) recovery in hydrometallurgical processes.

## 2. Materials and Methods

### 2.1. Materials

**Figure 1:**
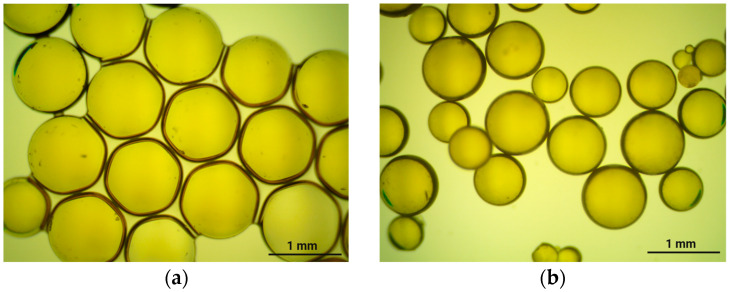
Optical microscopic images of KU-2-8 (**a**) and AV-17-8 (**b**) at 40× magnification.

The interpolymer system utilized KU-2-8 (H^+^ form) (Novokhim, Kharkiv, Ukraine), a strongly acidic cation exchanger with sulfonic acid functional groups, and AV-17-8 (OH^−^ form) (Azot, Cherkasy, Ukraine), a strongly basic anion exchanger with quaternary ammonium functional groups. Both resins, based on styrene-divinylbenzene copolymers, have particle size ranges of 0.30–1.25 mm. Simulated solutions were prepared in distilled water, containing 30 mg/L of Au(I) (derived from K[Au(CN)_2_], Sigma-Aldrich, Darmstadt, Germany) and 30 mg/L of Fe(II) (derived from K_4_[Fe(CN)_6_]·3H_2_O, Sigma-Aldrich, Germany). Desorption experiments were conducted using a solution containing 9% (*w/v*) thiourea and 2% (*v/v*) sulfuric acid to facilitate the formation of the positively charged thiocarbamide complex {Au[CS(NH_2_)_2_]}^+^. All chemicals used in the experiments were of analytical reagent grade, and deionized water with a resistivity of ~18.1 MΩ·cm was used throughout. Figure 1 demonstrates the optical microscopic images of KU-2-8 and AV-17-8 at 40× magnification.

### 2.2. Equipment

The optical microscopic images of KU-2-8 and AV-17-8 ion-exchange resins were obtained using a Levenhuk D320L microscope (Levenhuk, Inc., Tampa, FL, USA) at 40× magnification. Residual ion concentrations were determined using an inductively coupled plasma optical emission spectrometer (ICP-OES, iCAP PRO XP, Thermo Fisher Scientific, Loughborough, Leicestershire, UK) across a wavelength range of 167.021–852.145 nm. Fourier Transform Infrared (FTIR) spectroscopy (transmission mode) (Nicolet 5700, Thermo Fisher Scientific, Madison, WI, USA) was utilized to investigate changes in functional groups, with samples prepared by mixing with potassium bromide (KBr) and pressing into pellets, acquiring spectra in the range of 500–4000 cm^−1^ at 0.4 cm^−1^ resolution with 32 scans. TGA/DSC analysis were conducted using a TG 209 F3 instrument (NETZSCH, Selb, Bavaria, Germany).

### 2.3. Experimental Procedures

Sorption experiments were conducted using solutions containing 30 mg/L of Au(I) and Fe^2+^ ions over a 48-h period. Aliquots of 2 mL were collected at time intervals of 0.5, 2.5, 6, 24, and 48 h for analysis. The sorption efficiency (η) was calculated using the following equation:

(1) $$\eta =\frac{C_{o}-C_{e}}{C_{o}}\times 100\%$$

where C_o_ and C_e_ are initial and residual ion concentrations (mg/L), respectively.

The degree of polymer chain binding (θ) by the KU-2-8 (H^+^):AV-17-8 (OH^−^) interpolymer system was determined as the fraction of functional groups occupied by sorbed gold (Au^+^) and iron (Fe^2+^) ions, calculated using the following equation:

(2) $$\theta =\frac{\nu_{sorb}}{\nu }\times 100\%$$

where ν_sorb_ is moles of sorbed ions, and ν is total polymer mass (mol).

Desorption experiments were conducted over 72 h using a solution of 9% thiourea and 2% sulfuric acid as the eluent. Aliquots of 2 mL were collected at time intervals of 0.5, 2.5, 6, 24, 48, and 72 h for analysis. The desorption efficiency (R) was calculated using the following equation:

(3) $$R=\frac{C_{des}}{C_{sorb}}\times 100\%$$

where C_des_ is the desorbed ion concentration (mg/L), and C_sorb_ is the sorbed ion amount.

The desorption kinetics were mathematically evaluated using empirical models, specifically the pseudo-first-order (PFO) and pseudo-second-order (PSO) equations. These models were applied to the time-dependent desorption data, where q_t_ (mg/g) is the amount of ion desorbed per gram of polymer at time t (h), and q_e_ is the equilibrium desorption capacity. The PFO model is:

(4) $$q_{t}=q_{e}(1-e^{-k_{1}t})$$

where k_1_ is the PFO rate constant (h^−1^).

The PSO model is:

(5) $$q_{t}=\frac{k_{2}q_{e}^{2}t}{1+k_{2}q_{e}t}$$

where k_2_ is the PSO rate constant (g mg^−1^ h^−1^).

Model fitting was performed using nonlinear regression, and goodness-of-fit was assessed via the coefficient of determination (R^2^). For consistency with concentration data, q_t_ was calculated as (C_des_ × V)/m, where V is solution volume (mL) and m is polymer mass (g); however, since V/m is constant, proportional fitting to C_des_ yields equivalent kinetics.

The distribution coefficient (K_d_) of Au(I) and Fe(II) ions was calculated using the following equation [41]:

(6) $$K_{d}=\frac{Z_{B}}{B}\times \frac{V}{m}$$

where Z_B_—the amount of metal ions sorbed by the polymer; B—the amount of metal ions remaining in the solution; V—solution volume (in ml); m—polymer mass (in g).

The ion separation coefficient (β) was determined by dividing the distribution coefficients (K_d_) of Au(I) and Fe(II) ions, determined at the same values of solution volume (V) and polymer mass (m) according to the following equation:

(7) $$\beta =\frac{K_{d(Au)}}{K_{d(Fe)}}$$

## 3. Results

### 3.1. Sorption Kinetics

#### Sorption of Au(I) and Fe(II) Ions from a Gold-Iron Mixed Solution

**Figure 2:**
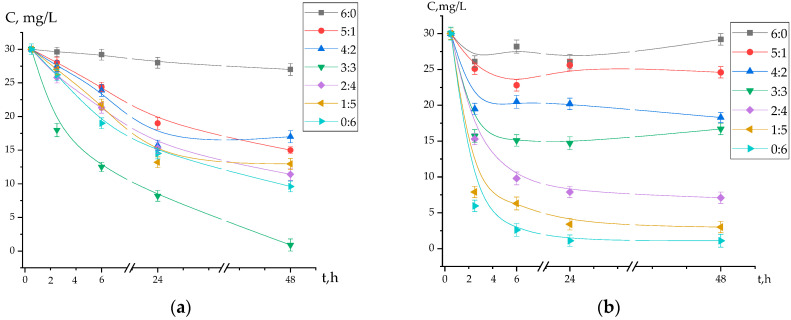
Concentrations of residual Au(I) (**a**) and Fe(II) (**b**) ions in a mixed solution after sorption by the interpolymer system KU-2-8:AV-17-8 as a function of time.

The interaction of the interpolymer system KU-2-8:AV-17-8 (X:Y) with Au(I) and Fe(II) ions, as shown in Figure 2, exhibits distinct time-dependent characteristics. Specifically, Figure 2a illustrates that at molar ratios of 6:0 and 0:6 (representing the initial polymers KU-2-8 and AV-17-8, respectively), the residual Au(I) concentration in the solution decreased from 30 mg/L to 27 mg/L and 10 mg/L, respectively, after 48 h of interaction. In contrast, at a molar ratio of 3:3 (representing the activated interpolymer system), the Au(I) concentration decreased significantly to approximately 1 mg/L after 48 h.

Similarly, Figure 2b shows that at a molar ratio of 6:0 (sole KU-2-8), the residual Fe(II) concentration in the solution decreased from 30 mg/L to 29 mg/L after 48 h. At a molar ratio of 0:6 (sole AV-17-8), the Fe(II) concentration decreased significantly to approximately 1.1 mg/L after 48 h.

Based on the data presented in Figure 2, the interpolymer system KU-2-8:AV-17-8 with a molar ratio of 3:3 exhibits high selectivity for Au(I) ions, as evidenced by the significant reduction in residual Au(I) concentration to approximately 1 mg/L after 48 h. In contrast, the anion-exchange resin AV-17-8 (molar ratio 0:6) demonstrates high selectivity for Fe(II) ions, with the residual Fe(II) concentration decreasing to approximately 1.1 mg/L after 48 h, as shown in Figure 2b.

The low performance (~10–20%) at 6:0 (sole KU-2-8) is anticipated, as KU-2-8, a cation exchanger, exhibits poor affinity for the anionic [Au(CN)_2_]^−^ and [Fe(CN)_6_]^4−^ complexes due to electrostatic repulsion, highlighting the synergistic enhancement in mixed ratios.

### 3.2. Polymer Chain Binding Degree (θ) of Gold Ions by the Interpolymer System

Investigating the polymer chain binding degree (θ) is critical for understanding the kinetics of ion sorption processes, particularly in interpolymer system such as KU-2-8(H^+^):AV-17-8(OH^−^). Here we explore the degree at which polymer chains bind gold ions from solution, such as auric cyanide, influenced by factors such as molar ratio and time. The binding degree provides insights into the efficiency and mechanism of ion exchange, with applications in gold recovery and material design. The polymer chain binding degree is defined as the speed at which active sites on polymer chains capture Au(I) ions ([Au(CN)_2_]^−^), typically measured as the change in ion concentration over time (mol/L·s). This process is governed by diffusion, surface reaction, and equilibrium.

**Figure 3:**
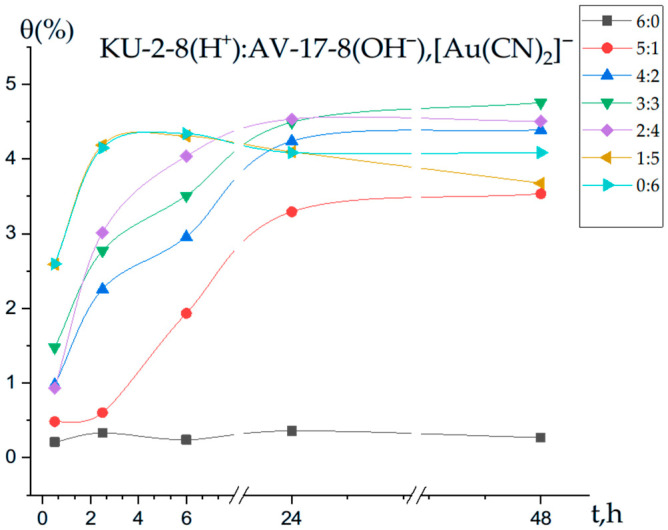
Degree of polymer chain binding of Au(I) ions by the interpolymer system KU-2-8(H^+^):AV-17-8(OH^−^) as a function of time.

This study investigates the binding capacity of the interpolymer system KU-2-8(H^+^):AV-17-8(OH^−^) for Au(I) ions at various molar ratios and time intervals, as shown in Figure 3. At the initial stage (0.5 h), the polymer chain binds only a limited amount of Au(I) ions (0.21%), indicating that the sorption process is in its early phase and the binding mechanisms are not yet fully activated.

In the case of the cation exchanger KU-2-8 alone (molar ratio 6:0), no significant changes in the degree of polymer chain binding of Au(I) ions were observed across all time intervals (0.5, 2.5, 6, 24, and 48 h). This suggests that, in the absence of the anion exchanger AV-17-8, the interpolymer system fails to achieve a highly charged state, limiting sorption efficiency. Conversely, for the anion exchanger AV-17-8 alone (molar ratio 0:6), the binding degree was relatively low at 0.5 h but increased steadily over time, reaching a maximum of 4.33% after 6 h. For the interpolymer system with a molar ratio of 3:3 (KU-2-8:AV-17-8), the binding degree of Au(I) ions was 1.47% at 0.5 h, indicating the onset of sorption by the polymer chains. Although the binding degree remained low initially, it gradually increased over time, reaching 4.75% after 48 h. This trend suggests that the sorption process in the 3:3 interpolymer system approaches an equilibrium state.

### 3.3. Polymer Chain Binding Degree (θ) of Iron Ions by the Interpolymer System

**Figure 4:**
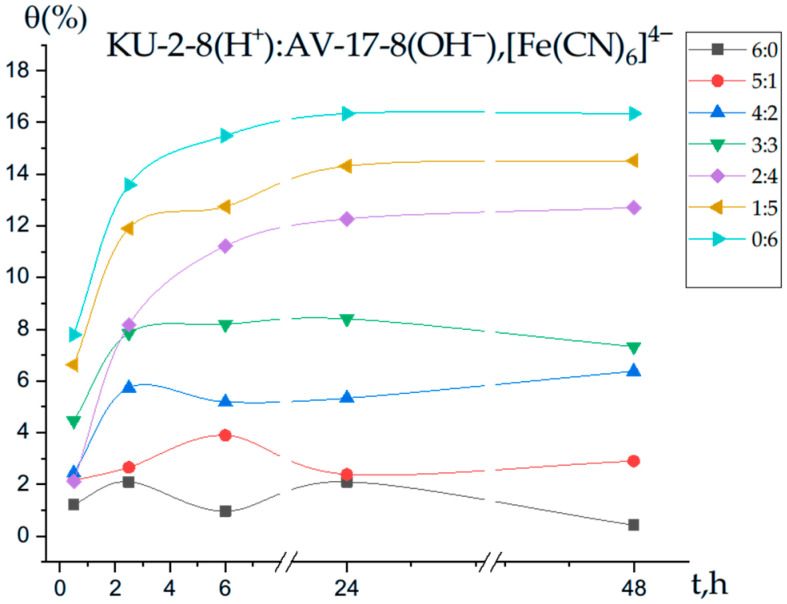
Degree of polymer chain binding of Fe(II) ions by the interpolymer system KU-2-8(H^+^):AV-17-8(OH^−^) as a function of time.

Figure 4 illustrates the degree of polymer chain binding of Fe(II) ions by the interpolymer system KU-2-8(H^+^):AV-17-8(OH^−^) as a function of time. For the cation exchanger KU-2-8(H^+^) alone (molar ratio 6:0), the binding degree of Fe(II) ions remains low across all time intervals (0.5, 2.5, 6, 24, and 48 h), with no significant increase, indicating a slow interaction process at this ratio. As the proportion of the anion exchanger AV-17-8(OH^−^) increases, the binding degree rises, reaching a maximum of 16.33% at a molar ratio of 0:6 (AV-17-8(OH^−^) alone) after 24 h. At this ratio, the binding degree increases to 15.48% after 6 h and remains stable at 16.33% after 48 h.

**Figure 5:**
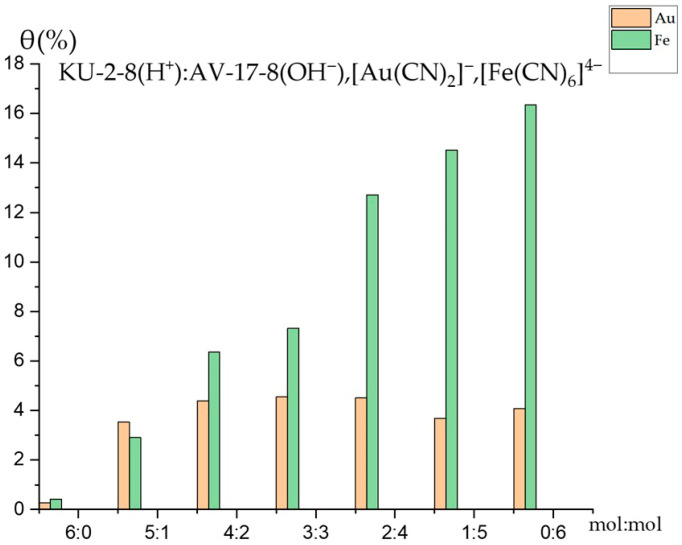
Comparison of binding degrees by the interpolymer system KU-2-8(H^+^):AV-17-8(OH^−^).

Figure 5 demonstrates the comparison of the polymer chain binding degrees of Au(I) and Fe(II) ions by the interpolymer system KU-2-8(H^+^):AV-17-8(OH^−^) under competitive sorption conditions. For the cation exchanger KU-2-8(H^+^) alone (molar ratio 6:0), after 48 h, the binding degree of Au(I) ions was 0.27%, while that of Fe(II) ions was 0.42%. Conversely, for the anion exchanger AV-17-8(OH^−^) alone (molar ratio 0:6), the binding degree of Au(I) ions was 4.08%, whereas that of Fe(II) ions was 16.33% after 48 h. These results indicate a significantly higher binding capacity of the AV-17-8(OH^−^) resin for Fe(II) ions compared to Au(I) ions under competitive conditions.

The results demonstrate that the binding degree of Fe(II) ions to the polymer chain is significantly higher than that of Au(I) ions, highlighting distinct interaction characteristics between the polymers and ions. As shown, the binding degree of Au(I) ions is markedly lower than that of Fe(II) ions across nearly all molar ratios, with Fe(II) ions achieving higher maximum values. Specifically, the binding degree for Fe(II) ions ranges from 0.41% to 16.34%, whereas for Au(I) ions, it ranges from 0.27% to 4.55%. These findings indicate that the interpolymer system KU-2-8(H^+^):AV-17-8(OH^−^) exhibits high activity and binding affinity for Fe(II) ions, while the binding affinity for Au(I) ions remains stable.

### 3.4. Competitive Sorption of the Gold and Iron Ions by the Interpolymer System KU-2-8(H+):AV-17-8(OH^−^)

**Figure 6:**
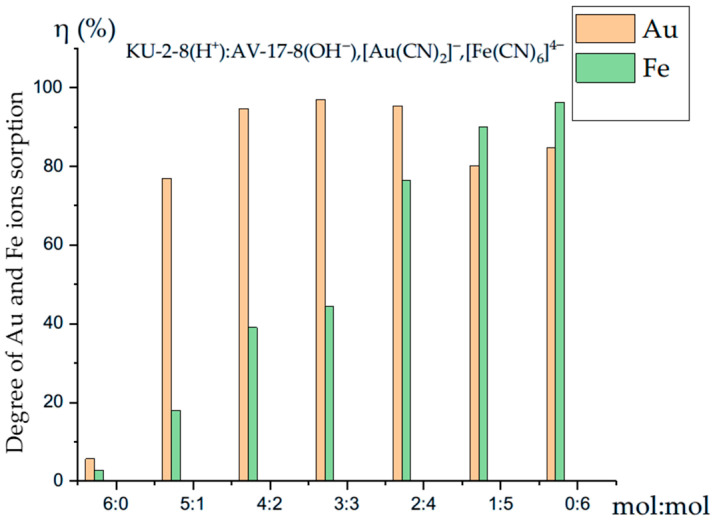
Comparative chart of competitive sorption of Au(I) and Fe(II) ions by the interpolymer system KU-2-8(H^+^):AV-17-8(OH^−^).

The variation in the rate of Au(I) and Fe(II) ions sorption based on the molar ratio is illustrated in Figure 6. This graph depicts the changes in the degree of separation of Au(I) and Fe(II) ions within the interpolymer system KU-2-8(H^+^):AV-17-8(OH^−^). The sorption of gold and iron ions on the individual ion exchanger KU-2-8(H^+^) (6:0) was 5.66% and 2.67%, respectively. The highest sorption values were recorded after 48 h of interaction at a ratio of 0:6, where the degree of sorption reached 96.33%.

At a polymer ratio of 5:1, the degree of Au(I) ion sorption was 77.00%, while the sorption of Fe(II) ions was significantly lower at only 18.00%. Following the interaction at 4:2, the degree of Au(I) ion sorption reached 94.67% after 48 h, whereas the degree of Fe(II) ion sorption during the same period was only 39.00%. In the interpolymer system KU-2-8(H^+^):AV-17-8(OH^−^) at ratios of 3:3 and 2:4, the maximum values of Au(I) ion sorption were recorded at 97.00% and 95.33%, respectively, while the sorption of Fe(II) ions at these ratios was less pronounced, at 44.33% and 76.33%. At a polymer ratio of 1:5, the degree of gold ion sorption was 80.13%.

Figure 6 illustrates that, across all ratios of the interpolymer system KU-2-8(H^+^):AV-17-8(OH^−^) (6:0, 5:1, 4:2, 3:3, and 2:4), the sorption efficiency of Au(I) ions exceeds that of Fe(II) ions. Conversely, at ratios of 1:5 and 0:6, the sorption efficiency of Fe(II) ions reaches 89.93% and 96.33%, respectively.

The competitive sorption study of Au(I) and Fe(II) ions by the interpolymer system KU-2-8(H^+^):AV-17-8(OH^−^) demonstrates that the maximum sorption capacity for Au(I) ions occurs at a ratio of 3:3, while the maximum sorption capacity for Fe(II) ions is observed at a ratio of 1:5.

### 3.5. Investigation of the Desorption of Au(I) and Fe(II) Ions

**Figure 7:**
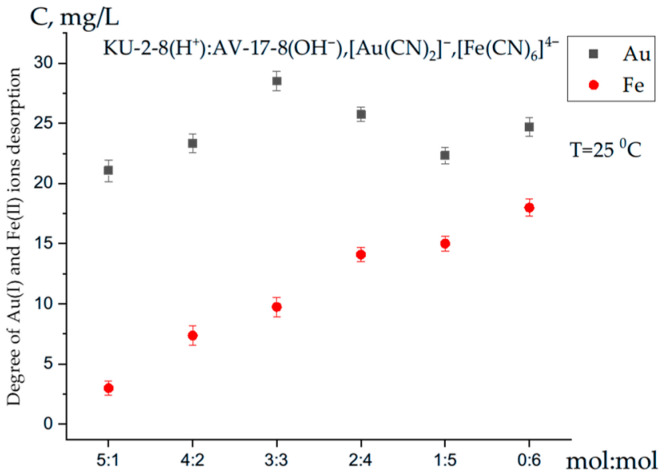
Desorption of Au(I) and Fe(II) ions from the interpolymer system KU-2-8(H^+^):AV-17-8(OH^−^).

The kinetics of Au(I) and Fe(II) ion desorption from the interpolymer system KU-2-8(H^+^):AV-17-8(OH^−^) are presented in Figure 7. The desorption efficiency, R (Au, Fe), was calculated using Equation (3). Interpolymer system, prepared at various molar ratios, were subjected to desorption in a solution containing thiourea and 2% (*v/v*) H_2_SO_4_ for 72 h. Desorption of Au(I) was carried out using a solution containing 9% (*w/v*) thiourea and 2% (*v/v*) sulfuric acid, which facilitated the formation of thiocarbamide complex, {Au[CS(NH_2_)_2_]}^+^, as shown in the reaction Equation (8):

RAu(CN)_2_ + 2CS(NH_2_)_2_ + H_2_SO_4_ → R_2_SO_4_ + Au[SC(NH_2_)_2_]_2_SO_4_ + 2HCN (8)

The concentrations of metal ions released from the polymer matrix into the acidic solution were determined using inductively coupled plasma optical emission spectrometer (ICP-OES). At a molar ratio of 3:3, the interpolymer system exhibited high desorption efficiency for Au(I) ions, achieving 97.28% (29.18 mg/L). In contrast, the desorption efficiency for Fe(II) ions was lower, reaching 62.28% (18.00 mg/L).

As shown in Figure 7, the desorption of Fe(II) ions increases proportionally with the anion exchanger content, rising from 3.00 mg/L to 18.00 mg/L. The desorption of Au(I) ions exhibits a more complex pattern. The maximum concentration of desorbed Au(I) ions increases from 21.08 mg/L at a molar ratio of 5:1 to 29.18 mg/L at an equimolar ratio of 3:3 in the KU-2-8(H^+^):AV-17-8(OH^−^) interpolymer system. At the 5:1 molar ratio, the desorbed concentrations were 21.08 mg/L for Au(I) ions and 3.00 mg/L for Fe(II) ions, indicating significantly higher desorption efficiency for Au(I) ions. The highest desorption values were observed at a 4:2 molar ratio, with concentrations of 23.34 mg/L for Au(I) ions and 7.36 mg/L for Fe(II) ions.

With increasing anion exchanger content in the KU-2-8(H^+^):AV-17-8(OH^−^) interpolymer system, desorption efficiency gradually increases. At an equimolar ratio of 3:3, the desorption of Au(I) ions reaches a maximum concentration of 29.18 mg/L, corresponding to 97.28% desorption efficiency. These findings indicate that the interpolymer system exhibits high desorption activity for Au(I) ions, with efficiency modulated by the molar ratio of the ion exchangers.

The high desorption efficiency of Au(I) ions at a 3:3 molar ratio strongly correlates with their previously reported maximum sorption efficiency at the same ratio, suggesting a close relationship between sorption and desorption processes in the KU-2-8(H^+^):AV-17-8(OH^−^) interpolymer system. In contrast, the desorption efficiency of Fe(II) ions was significantly lower, reaching a maximum of 60.4% (18.12 mg/L) at the 0:6 molar ratio. This lower efficiency suggests that Fe(II) ions form stronger coordination bonds with the functional groups of the interpolymer system compared to Au(I) ions.

At a molar ratio of 2:4, the desorption concentrations were 25.76 mg/L for Au(I) ions and 14.10 mg/L for Fe(II) ions, confirming higher desorption efficiency for Au(I) ions. At a 1:5 molar ratio, the desorption concentration of Au(I) ions decreased to 22.32 mg/L. This reduction may be attributed to the swelling behavior of the polymer network in an aqueous medium. During the initial swelling phase, the polymer network expands, facilitating the release of Fe(II) ions. However, as swelling progresses, the release of Fe(II) ions bound within the polymer network slows, reducing their desorption rate. These results demonstrate that the desorption of Au(I) and Fe(II) ions by the KU-2-8(H^+^):AV-17-8(OH^−^) inter-polymer system is directly influenced by the molar ratio of the ion exchangers.

#### 3.5.1. Mathematical Evaluation of Desorption Kinetics

To further evaluate the desorption kinetics, time-dependent data for Au(I) ions at the optimal equimolar ratio (3:3) were fitted to empirical models (Equations (4) and (5)). The desorption process was monitored at time intervals of 0.5, 2.5, 6, 24, 48, and 72 h, with desorbed concentrations (C_des_, mg/L) reaching equilibrium at approximately 28.5 mg/L (corresponding to q_e_ ≈ 28.5 mg/g, assuming standard V/m ratios). The experimental data show rapid initial desorption followed by a plateau, characteristic of chemisorption processes.

The kinetic parameters and goodness-of-fit are summarized in Table 1. The pseudo-second-order model provided an excellent fit (R^2^ = 0.999), with q_e_ = 28.49 mg/g and k_2_ = 0.050 g mg^−1^ h^−1^, outperforming the pseudo-first-order model (R^2^ = 0.936, q_e_ = 27.08 mg/g, k_1_ = 0.937 h^−1^). This suggests that the desorption rate is controlled by chemisorption mechanisms, involving electron sharing or exchange between Au(I) complexes and the eluent (thiourea/H_2_SO_4_), rather than simple physical diffusion. The higher R^2^ for the pseudo-second-order model aligns with similar studies on ion exchange desorption, indicating valence forces in the rate-limiting step.

#### 3.5.2. Ion Exchange Mechanisms

The sorption of Au(I) and Fe(II) cyanide complexes primarily may occur via anion exchange on AV-17-8 (quaternary ammonium sites, R^+^-OH^−^), as both are anionic. KU-2-8 (sulfonic acid sites, R-SO_3_H) contributes minimally to direct exchange due to Donnan exclusion (also known as the Gibbs-Donnan effect [42]) but facilitates mutual activation through remote proton donation, boosting overall selectivity.

For standalone AV-17-8 (anion exchange):

R^+^-OH^−^ + [Au(CN)_2_]^−^ ⇌ R^+^-[Au(CN)_2_]^−^ + OH^−^ (9)

R^+^-OH^−^ + ¼[Fe(CN)_6_]^4−^ ⇌ R^+^-¼[Fe(CN)_6_]^4−^ + OH^−^ (10)

For standalone KU-2-8 (cation exchange, negligible for anions), there is no significant reaction due to repulsion:

R-SO_3_^−^ H^+^ + [Au(CN)_2_]^−^ → no exchange (11)

In the KU-2-8:AV-17-8 interpolymer system, remote interaction promotes:

R-SO_3_^−^ H^+^ (KU-2-8) + R^+^-OH^−^ (AV-17-8) → R-SO_3_^−^…H-O^+^-R (activated pair) + H_2_O (12)

This generates localized acidity, favoring [Au(CN)_2_]^−^ uptake (Equation (9)) over [Fe(CN)_6_]^4−^ (Equation (10)) by reducing steric hindrance and enhancing β_Au/Fe_ via higher K_d_ for monovalent anions (3233 mL/g vs. ~80 mL/g for Fe). Desorption uses thiourea/H_2_SO_4_:

R^+^-[Au(CN)_2_]^−^ + [SC(NH_2_)_2_H]^+^ + HSO_4_^−^ → R^+^-HSO_4_^−^ + [Au(SC(NH_2_)_2_)_2_]^+^ (13)

### 3.6. Fourier Transform Infrared (FTIR) Spectroscopy Studies

To investigate the interaction characteristics of the KU-2-8 (H^+^):AV-17-8 (OH^−^) interpolymer system with Au(I) and Fe(II) ions, samples exhibiting the highest ion sorption were analyzed using Fourier Transform Infrared (FTIR) spectroscopy.

**Figure 8:**
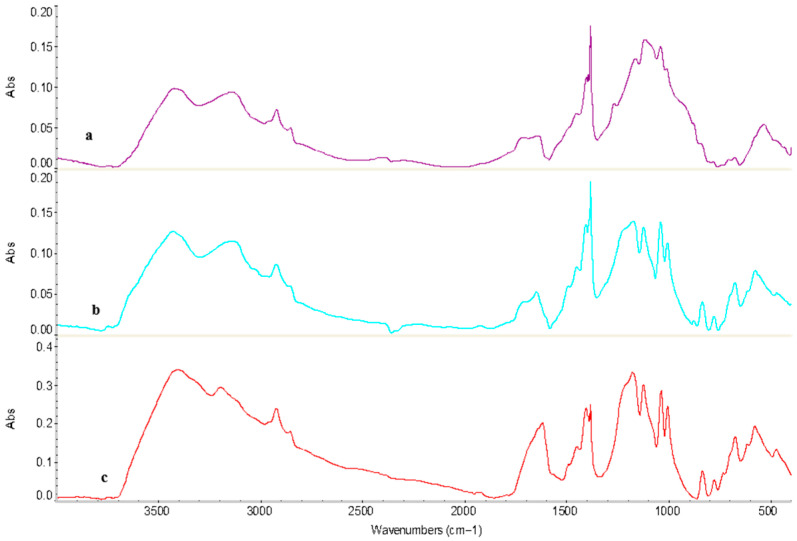
FTIR spectra of initial KU-2-8(H^+^) (**a**), KU-2-8(H^+^) from the interpolymer system KU-2-8(H^+^):AV-17-8(OH^−^) (3:3) after sorption (**b**), and KU-2-8(H^+^) (3:3) after desorption (**c**).

For the initial KU-2-8(H^+^) (Figure 8a), key peaks include: 3426.0 cm^−1^ (O–H stretching in sulfonic acid groups and adsorbed water [43]), 2923.6 cm^−1^ (asymmetric C–H stretching in aliphatic chains [44]), 1637.0 cm^−1^ (aromatic C=C stretching [45]), ~1450 cm^−1^ (C–H deformation vibrations of the methylene (–CH_2_−) groups in the polymer backbone, indicating the aliphatic components of the styrene-divinylbenzene copolymer structure [44], (p. 83)), 1164.5 cm^−1^ (S=O asymmetric stretching in –SO_3_H [46]), 1117.9 cm^−1^ (C–O–C or in-plane C–H bending [47]), 1039.4 cm^−1^ (S=O symmetric stretching [46], (p. 394)), and lower-frequency bands such as 675.7 cm^−1^ and 576.9 cm^−1^ (out-of-plane C–H bending in aromatic rings [44], (p. 85)). These assignments align with reported spectra for sulfonated polystyrene resins [42,43,44,45,46,47], confirming the integrity of the functional groups without significant impurities.

Following the sorption process in the KU-2-8(H^+^):AV-17-8(OH^−^) interpolymer system at a 3:3 molar ratio, FTIR spectroscopy revealed minor shifts in absorption bands and the emergence of new bands (Figure 8b). In the high-frequency region, absorption bands associated with hydroxyl (–OH) groups shifted from 3426.0 cm^−1^ and 3237.5 cm^−1^ in the unmodified cation exchanger to 3430.5 cm^−1^ and 3135.4 cm^−1^, respectively, indicating the formation of coordination bonds between Au(I) and Fe(II) ions and hydroxyl groups. The C–H stretching vibrations, observed at 2923.6 cm^−1^ before sorption, remained nearly unchanged at 2925.3 cm^−1^ after sorption and 2925.0 cm^−1^ after desorption, suggesting minimal alteration of hydrocarbon bonds during interactions with Au(I) and Fe(II) ions. Additionally, Figure 8c shows an increase in intensity around 1637 cm^−1^ (shifted to ~1610 cm^−1^ post-desorption), associated with aromatic C=C stretching, but potentially overlapping with emerging C=O vibrations from partial oxidation. This could arise from oxygen interactions with the swollen polymer structure during the desorption process in acidic media, leading to minor carbonyl formation.

After desorption (Figure 8c), some absorption bands returned to values close to those of the initial state, but complete recovery was not observed. Bands at 1177.4 cm^−1^, 1124.3 cm^−1^, and 1036.4 cm^−1^ indicate partial retention of structures formed during sorption. Residual functional groups were evident from bands at 1005.8 cm^−1^ (C–C stretching in the polymer backbone [48]), 833.6 cm^−1^, and 775.9 cm^−1^ (out-of-plane C–H bending in aromatic rings [49]), suggesting stabilization or additional cross-linking of the resin molecules post-sorption. Peaks at 674.6 cm^−1^, 577.5 cm^−1^, and 472.1 cm^−1^ confirm the persistence of metal-ligand vibrations (e.g., Au–O or Fe–O/S from coordination with sulfonate groups [50,51]), indicating stable ionic interactions with Au(I) and Fe(II).

**Figure 9:**
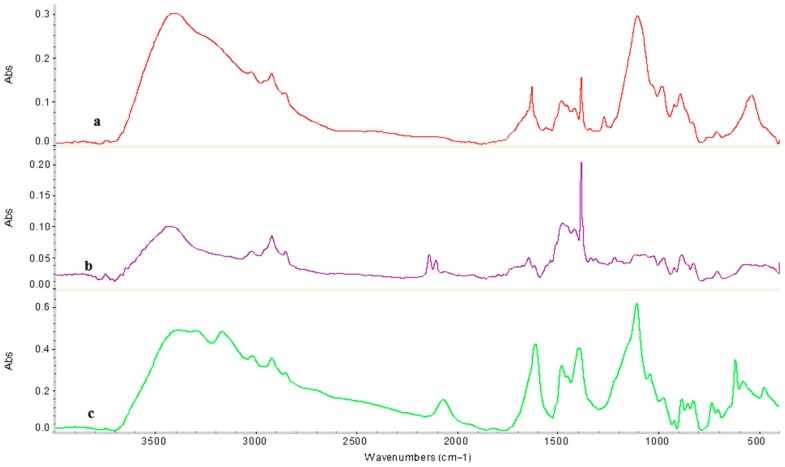
FTIR spectra of initial AV-17-8 (OH^−^) (**a**), AV-17-8 (OH^−^) from interpolymer system KU-2-8(H+):AV-17-8 (OH^−^) (3:3) after sorption (**b**) and AV-17-8 (OH^−^) (3:3) after desorption (**c**).

Similarly, for the initial AV-17-8(OH^−^) (Figure 9a), peaks include: broad band ~3400 cm^−1^ is O–H stretching in quaternary ammonium hydroxide and water [52]; 2924.2 cm^−1^ and 1481.3 cm^−1^ (C–H stretching and bending in alkyl chains [53]); 1632 cm^−1^ is attributed to aromatic C=C stretching vibrations [54], ~1480–1550 cm^−1^ is stretching vibrations of quaternary ammonium (–N^+^(CH_3_)_3_) groups, confirming the resin’s functional sites [44], (p. 101); ~1380 cm^−1^ is symmetric C–H bending of methyl groups in the quaternary ammonium structure; ~1200–1300 cm^−1^ is C–N stretching or aromatic C–H in-plane bending from the polystyrene-divinylbenzene backbone [55]; ~700–900 cm^−1^ is out-of-plane C–H bending of the aromatic rings, typical of monosubstituted benzene in polystyrene [56]. These match literature data for anion exchanger with trimethylammonium groups [52,53,54,55,56], verifying the sample’s purity and structural stability.

The observed shifts in band positions are attributed to ion coordination mechanisms: blueshifts (e.g., S=O from 1164.5 to 1177.3 cm^−1^) suggest bond strengthening due to reduced electron density from metal ion binding [57], while redshifts (e.g., C–N from 1270.5 to 1219.2 cm^−1^ in AV-17-8) indicate weakening from electron donation or hydrogen bonding changes.

Post-sorption, new absorption bands appeared at 2140.4 cm^−1^ and 2108.3 cm^−1^, indicative of C≡N stretching vibrations from the cyano ligands in sorbed [Au(CN)_2_]^−^ and [Fe(CN)_6_]^4−^ complexes, shifted due to metal-ligand coordination with the resin’s functional groups [58,59]. Changes in bands at 1219.2 cm^−1^, 1074.2 cm^−1^, and 564.6 cm^−1^ suggest partial restoration of C–N, C–O, and bonds associated with heavy atoms after sorption. Following desorption (spectrum c), some original bands reappeared, but peaks at 3384.9 cm^−1^, 2071.5 cm^−1^, 580.3 cm^−1^, and 476.9 cm^−1^ persisted, indicating incomplete removal of sorbed Au(I) and Fe(II) ions. This suggests that the desorption process is partially irreversible.

Comparison of Figure 9a,c reveals increased line widths post-desorption, particularly for O–H (~3400 cm^−1^) and C=C (~1600 cm^−1^) bands, suggesting enhanced disorder due to residual swelling or incomplete recovery of the polymer structure [60]. This may result from water retention or ion-induced conformational heterogeneity in the resin.

New absorption bands at 3171.1 cm^−1^ (O–H in oxidized groups such as carboxylic acids [61]), 3019.3 cm^−1^ (aromatic C–H stretching vibrations [62]), 922.5 cm^−1^ (vinyl C–H bending indicating chain flexibility changes), 735.8 cm^−1^ (C–S stretching suggesting cross-linking or stabilization [63]), and 618.5 cm^−1^ (metal-coordination vibrations) emerged. These indicate structural changes related to partial oxidation (from oxygen exposure during processes), disordering (increased heterogeneity in polymer chains), and cross-linking (ion-induced stabilization), alongside coordination interactions. These findings demonstrate that interactions with Au(I) and Fe(II) ions induce significant, stable modifications in the AV-17-8(OH^−^) anion exchanger, affecting its sorption properties.

Bands at 675.7 cm^−1^ (intensified from ~673 cm^−1^ in pristine KU-2-8) and 576.9 cm^−1^ (shifted from ~574 cm^−1^ in pristine AV-17-8), associated with C–S and C–N bending modes in the polymer backbone, exhibit enhanced intensity post-sorption, indicating stabilization by metal-ion interactions (e.g., Au(I) coordination to sulfonic/quaternary groups) rather than de novo formation. These fingerprint region features, common in polystyrene-based resins, underscore conformational adjustments without introducing entirely new vibrational signatures.

### 3.7. Thermogravimetric and Differential Scanning Calorimetric Analyses of the Interpolymer System KU-2-8(H^+^):AV-17-8(OH^−^) (3:3) and Its Complexes

**Figure 10:**
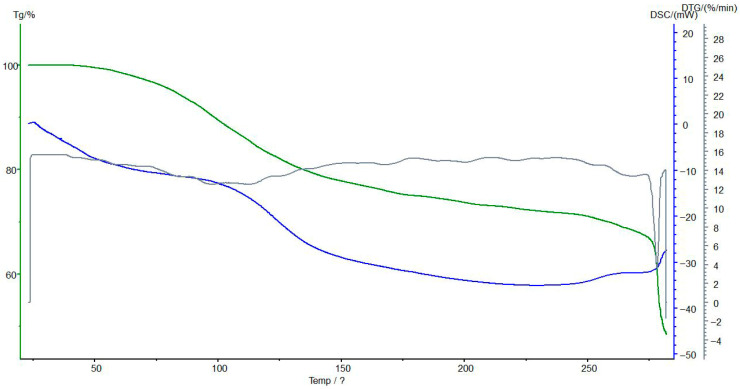
The TG (green line), DSC (blue line), and DTG (gray line) analyses of the initial KU-2-8(H^+^). Legend: Mass loss (%) vs. temperature (°C); reduced mass loss up to 100 °C post-sorption indicates enhanced thermal stability due to metal coordination. Conditions: 10 °C/min heating rate under N_2_ flow.

The TG/DSC analyses of the KU-2-8 (H^+^):AV-17-8 (OH^−^) interpolymer system at a 3:3 molar ratio, both in its native form and complexed with Au(I) ions were used to investigate the thermal stability, decomposition behavior, and phase transitions of the interpolymer system and its ion complexes, providing insights into the structural and chemical changes induced by ion sorption. Figure 10 presents the results of TG/DSC analysis of initial KU-2-8(H^+^) (before sorption).

**Figure 11:**
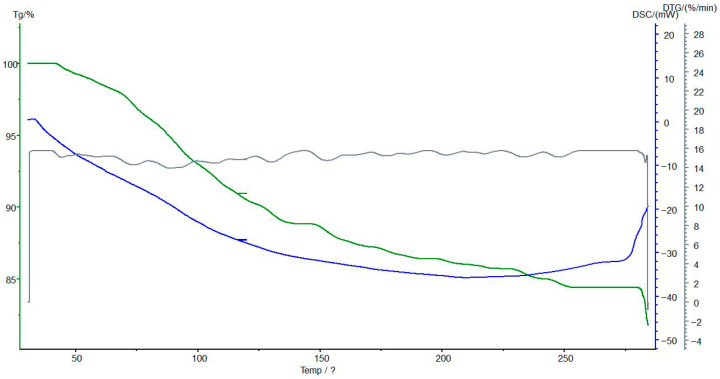
The TG (green line), DSC (blue line), and DTG (gray line) analyses of KU-2-8(H^+^) from the interpolymer system KU-2-8(H^+^):AV-17-8(OH^−^) (3:3) with Au(I) ions. Legend: Mass loss (%) vs. temperature (°C); reduced mass loss up to 100 °C post-sorption indicates enhanced thermal stability due to metal coordination. Conditions: 10 °C/min heating rate under N_2_ flow.

Figure 11 presents the results of TG/DSC analyses of KU-2-8(H^+^) from the interpolymer system KU-2-8(H^+^):AV-17-8(OH^−^) (3:3) with sorbed Au(I) ions.

TG analysis reveals an initial mass loss of up to 100 °C in the unsorbed state, attributable to moisture evaporation. Post-sorption, this mass loss range narrows, and enhanced thermal stability is observed between 150–200 °C, likely due to the stabilizing effect of gold and iron ion complexation. Despite desorption, the material does not fully revert to its initial thermal profile, indicating partial irreversibility of the sorption process, as evidenced by residual mass changes.

DSC analysis shows an endothermic peak up to 100 °C before sorption, consistent with moisture loss, which persists post-sorption but diminishes in intensity by 150 °C, suggesting improved thermal stability due to altered hydration dynamics. No new exothermic peaks emerge at 200–250 °C before or after sorption, supporting the occurrence of significant structural modifications during ion interaction. These findings suggest that KU-2-8(H^+^) undergoes functional changes upon possible binding Au(I) ions, potentially enhancing its sorption capacity, though further quantitative analysis.

**Figure 12:**
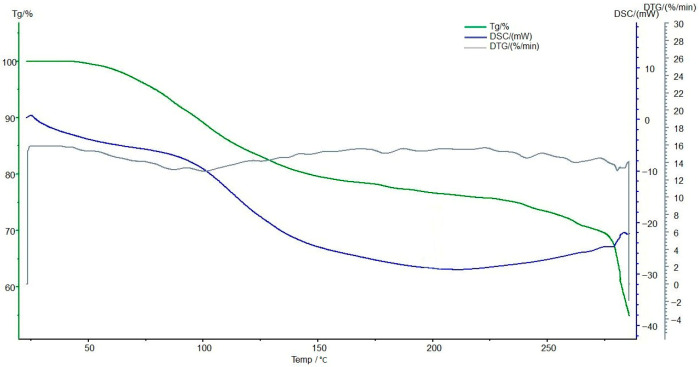
The TG (green line), DSC (blue line), and DTG (gray line) analyses of the initial AV-17-8(OH^−^). Legend: Heat flow (mW/mg) vs. temperature (°C); endothermic peaks at ~80–120 °C shift post-sorption, reflecting stabilized polymer matrix. Conditions: 10 °C/min heating rate under N_2_ flow.

Figure 12 presents the TG/DSC analysis of the initial anion exchanger AV-17-8(OH^−^) before sorption, revealing some alterations in its thermal properties.

**Figure 13:**
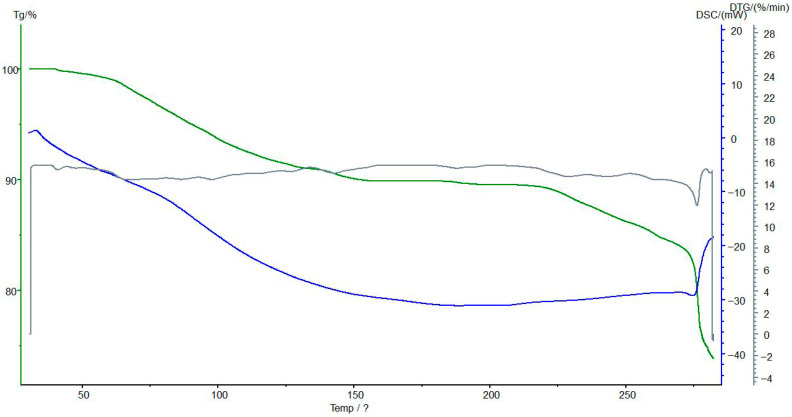
The TG (green line), DSC (blue line), and DTG (gray line) analyses of AV-17-8(OH^−^) from the interpolymer system KU-2-8(H^+^):AV-17-8(OH^−^) (3:3) with Au(I) ions. Legend: Heat flow (mW/mg) vs. temperature (°C); endothermic peaks at ~80–120 °C shift post-sorption, reflecting stabilized polymer matrix. Conditions: 10 °C/min heating rate under N_2_ flow.

Figure 13 presents the TG/DSC analysis of the anion exchanger AV-17-8(OH^−^) after sorption, revealing the alterations in its thermal properties. TG analysis indicates that the unsorbed material exhibits the greatest weight loss in the 150–200 °C range, likely corresponding to the thermal decomposition of polymer functional groups. Post-sorption, the rate of weight loss decreases, and the onset of decomposition shifts to higher temperatures, suggesting enhanced structural stability due to ion binding. DSC analysis confirms an endothermic process in the 150–200 °C range before sorption, which diminishes in intensity afterward, indicative of improved thermal stability possibly linked to reduced moisture or altered polymer-ion interactions. No new exothermic peaks are observed at 200–250 °C post-sorption, supporting the occurrence of significant structural modifications. These results suggest that sorption of metal ions subtly increases the thermal stability of AV-17-8(OH^−^) and reduces its decomposition rate, supporting its potential application in metal ion sorption and processing.

### 3.8. Determination of Ion Distribution and Separation Coefficients

The distribution coefficients (K_d_) for Au(I) and Fe(II) ions were calculated using Equation (6), based on the sorption data obtained after 48 h of interaction. For standardization, a solution volume (V) of 100 mL and polymer mass (m) of 1 g were assumed, yielding K_d_ values in mL/g. The separation coefficients (α) were determined using Equation (7) as the ratio of K_d_ for Au(I) to K_d_ for Fe(II). These parameters provide quantitative insights into the selectivity of the interpolymer system KU-2-8(H^+^):AV-17-8(OH^−^) for Au(I) ions in the presence of Fe(II) ions under competitive conditions. Table 2 summarizes the calculated K_d_ values for Au(I) and Fe(II) ions, along with the corresponding separation coefficients (β), at various molar ratios.

The results indicate that the distribution coefficient for Au(I) ions reaches its maximum value of 3233.3 mL/g at the equimolar ratio of 3:3, reflecting the highest sorption affinity at this composition. In contrast, the K_d_ for Fe(II) ions increases with higher proportions of AV-17-8(OH^−^), peaking at 2627.3 mL/g for the 0:6 ratio (pure AV-17-8(OH^−^)). The separation coefficient (β) is highest at the 3:3 ratio (40.62), demonstrating superior selectivity for Au(I) over Fe(II) ions, which aligns with the observed maximum sorption efficiency of 97.0% for Au(I) at this ratio.

As the molar ratio shifts toward higher AV-17-8(OH^−^) content (e.g., 1:5 and 0:6), β decreases significantly below 1, indicating a reversal in selectivity favoring Fe(II) ions. This behavior underscores the synergistic remote interaction effect in the interpolymer system, where the balanced 3:3 ratio optimizes electrostatic and conformational changes in the polymer matrix for preferential Au(I) binding. These coefficients confirm the system’s potential for selective gold recovery in hydrometallurgical processes, with the 3:3 composition offering the best balance of distribution and separation performance.

### 3.9. Langmuir Isothermal Model

The adsorption isotherms were determined using the Langmuir model, which assumes monolayer adsorption on a homogeneous surface with uniform binding sites and constant adsorption energy across all sites. According to this model, the amount of metal ions adsorbed onto the ion-exchange resin increases hyperbolically with the equilibrium concentration in the liquid phase, approaching a maximum saturation value [64]. This relationship can be described by the following linearized Equation (14):

(14) $$\frac{1}{Q_{e}}=\;\frac{1}{Q_{m}K_{L}C_{e}}+\;\frac{1}{Q_{m}}$$

where Q_e_ is the adsorption capacity adsorbed at equilibrium, Q_m_ is the maximum adsorption capacity (anions mg/g of resin), k_L_ is the Langmuir adsorption constant (L/mg), and C_e_ is the equilibrium concentration of the adsorbate (mg/L).

**Figure 14:**
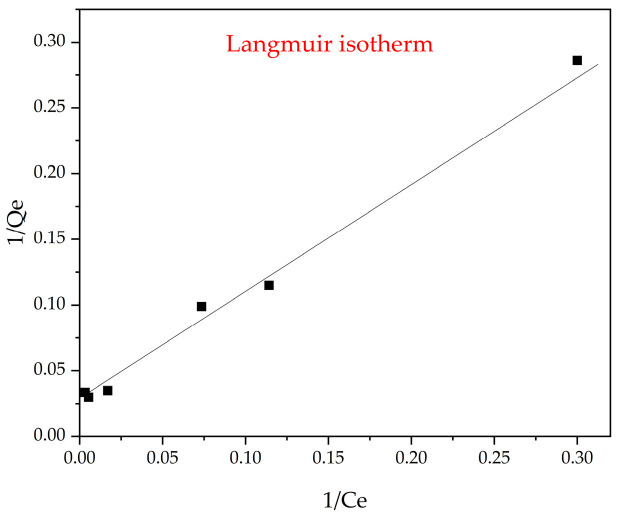
Langmuir isothermal model for the interpolymer system “KU-2-8:AV-17-8” (3:3) Au(I). The black squares (■) represent the experimental data points, and the solid line corresponds to the Langmuir isotherm fit.

The current work applies the Langmuir isotherm to characterize the adsorptive performance of the interpolymer system KU-2-8:AV-17-8 at a 3:3 molar ratio with respect to Au(I) ions, as illustrated in Figure 14. This sorbent configuration demonstrated a strong affinity for Au(I) uptake from solution, and its conformity to the Langmuir model—which assumes monolayer adsorption on a homogeneous surface with uniform binding sites and constant adsorption energy—confirms its suitability for targeted recovery applications.

Evaluation of the Langmuir isotherms (Figure 14) for the KU-2-8:AV-17-8 (3:3) interpolymer system toward Au(I) ions provided key insights into its sorption dynamics. The interpolymer system demonstrated strong adherence to the Langmuir model, which assumes monolayer adsorption on a homogeneous surface with uniform binding sites and constant adsorption energy, highlighting its effectiveness as a sorbent for Au(I) recovery. This conformity suggests that this interpolymer system offers high-performance sorption with a well-defined maximum capacity across certain polymer ratio.

## 4. Conclusions

In recent decades, the demand for precious metals has surged significantly, primarily due to their indispensable role as key components in advanced technologies, electronics, nanotechnology, catalysis, and jewelry. However, the extraction of gold from secondary sources, such as industrial effluents containing complex cyanide complexes (e.g., [Au(CN)_2_]^−^ coexisting with [Fe(CN)_6_]^4−^), remains challenging due to the chemical stability of these species, low selectivity of traditional methods, and environmental concerns associated with solvent extraction and precipitation.

Ion exchange resins are widely employed across scientific, technological, and industrial fields for metal ion recovery. Their application in sorption processes is particularly pertinent, as these materials exhibit high exchange capacity, chemical stability, and resistance to degradation in harsh aqueous environments. A broad array of commercial ion exchangers, such as the strongly acidic cation resin KU-2-8 (H^+^ form) and strongly basic anion resin AV-17-8 (OH^−^ form), are produced annually for precious metal recovery. Yet, experimental evidence reveals that many such sorbents suffer from limited efficiency, poor selectivity in the presence of competing ions (e.g., Fe(II)), and suboptimal desorption performance, restricting their widespread adoption.

Polystyrene-divinylbenzene-based resins with sulfonic acid and quaternary ammonium functional groups represent highly selective polymer structures capable of binding metal complexes. This enables effective sorption of precious metal anions, such as Au(I), even in multicomponent solutions. Non-ionic or homogeneously charged polymers incapable of ionization and dissociation do not engage in remote interactions, per the principles governing interpolymer phenomena in aqueous media. Consequently, the remote interaction of oppositely charged exchangers with distinct structures promotes their mutual activation and functionalization.

Prior studies have demonstrated that remote interactions in interpolymer systems lead to marked enhancements in electrochemical, conformational, and sorption properties. The formation of uncompensated functional groups, stabilized by intramolecular forces, arises from these interactions; resultant electrochemical and conformational shifts in the polymer matrix substantially boost the system’s overall sorption capacity. In such setups, the “long-range effect” yields exceptional selectivity toward target ions, as evidenced here by the KU-2-8:AV-17-8 (3:3) system achieving 97.0% Au(I) sorption efficiency (K_d_ = 3233.3 mL/g) and a separation coefficient β_Au/Fe_ = 40.62 after 48 h, with 98.0% desorption using thiourea/sulfuric acid. FTIR analysis confirmed stable Au(I) interactions via band shifts (e.g., 1273.5 to 1292.9 cm^−1^) and new absorptions (e.g., 3171.1 cm^−1^), while TGA/DSC revealed improved thermal stability (reduced mass loss up to 100 °C).

This research validates the interpolymer system’s potential for sustainable gold recovery in hydrometallurgy, offering a cost-effective, eco-friendly alternative that minimizes waste and enhances selectivity over interferents such as Fe(II). Future work could extend this approach to other precious metals, optimizing ratios for pilot-scale implementation and broader industrial viability.

## Figures and Tables

**Table 1:** Kinetic Parameters for Au(I) Desorption Models.

Model	q_e_ (mg/g)	Rate Constant	R^2^
Pseudo-first-order	27.08	k_1_ = 0.937 h^−1^	0.936
Pseudo-second-order	28.49	k_2_ = 0.050 g mg^−1^ h^−1^	0.999

**Table 2:** Distribution coefficients (K_d_) for Au(I) and Fe(II) ions and separation coefficients (β) at different molar ratios of the KU-2-8(H^+^):AV-17-8(OH^−^) interpolymer system.

Molar Ratio (KU-2-8:AV-17-8)	K_d_ Au (mL/g)	K_d_ Fe (mL/g)	β (K_d_ Au/K_d_ Fe)
6:0	6.0	2.7	2.22
5:1	334.8	22.0	15.22
4:2	1775.0	64.0	27.73
3:3	3233.3	79.6	40.62
2:4	2042.9	322.5	6.33
1:5	403.0	893.0	0.45
0:6	200.0	2627.3	0.08

## Data Availability

The original contributions presented in this study are included in the article. Further inquiries can be directed to the corresponding author.
